# Midwife students’ attitudes towards violence against women: A pilot study

**DOI:** 10.18332/ejm/193603

**Published:** 2024-10-14

**Authors:** Marika Merits, Kaire Sildver, Katrin Klein, Triin Lillsoo

**Affiliations:** 1 Department of Midwifery, Health Education Centre, Tallinn Health Care College, Tallinn, Estonia

**Keywords:** attitude, violence against women, midwife student, pilot study

## Abstract

**INTRODUCTION:**

The United Nations define violence against women (VAW) as any gender-based violence that causes mental, physical, or economic harm and restricts freedom. The topic has not been studied before in the context of the midwifery curriculum in Estonia. The purpose of the study is to investigate the attitudes of midwife students towards VAW.

**METHODS:**

This pilot study followed a mixed-methods approach. The study population consisted of 77 midwifery students at Tallinn Health Care College from 2022–2023. The online survey was distributed to all Tallinn Health Care College midwifery students. The pilot study is used to validate the questionnaire and obtain initial information.

**RESULTS:**

The results show that the midwife students of this study have personal experiences and exposures to different types of violence, and some midwife students had sociocultural misconceptions about VAW. Midwife students have limited knowledge of the impact of violence on women’s health and the legislation regarding violence. Midwife students stated that they would not be able to properly help the woman as a victim, as they lack knowledge, skills, and experience, which is an unfortunate factor.

**CONCLUSIONS:**

The present pilot study showed that midwife students’ attitudes towards VAW need improvement. The study raises the need to investigate midwife students’ attitudes towards VAW with a larger sample, better-designed method, and validated questionnaire. Topics related to violence should be included in the midwifery curriculum, along with developing practical skills.

## INTRODUCTION

The United Nations define violence against women (VAW) as any gender-based violence that causes or is likely to cause mental, physical, or sexual harm, including acts of intimidation and coercion or deprivation of liberty, whether in public or private life. VAW can include mental/emotional, physical, sexual, cultural/spiritual, and financial violence, and a wide range of controlling, coercive and intimidating behaviors^1^. A report compiled by the WHO, which analyzed data from 161 countries from 2000 to 2018, found that globally, 30% of women aged 15–50 years have experienced physical, mental, or sexual violence by an intimate partner or non-partner^2^. VAW is internationally described as a serious global public health problem with severe consequences, not only for the woman herself but also for her children. Besides individual suffering, VAW causes serious short- and long-term mental health, physical health, and sexual and reproductive health problems for victims^2^. A survey was conducted among Estonian healthcare workers in 2014, which revealed that blaming the victim prevails among healthcare workers, where 28–79% believe that the woman herself provokes VAW with her behavior, 91–94% of the respondents mention time as an obstacle to helping the victim lack, and 86–97% of the respondents point to the lack of special preparation as the main reason^3^.

Misconceptions and a lack of professional skills to help women as victims can normalize VAW in the eyes of healthcare professionals, leading to victim blaming instead of helpful and supportive actions^4^. However, health professionals, nurses, and midwives are in a unique position to identify and assist victims of VAW as they are often their first point of contact in the healthcare system^5-8^. The willingness of healthcare students to recognize the signs of violence and help the victim is influenced by their attitudes^9-11^. Students may be aware of the importance of teaching about violence, but their attitudes and knowledge levels are not sufficient to help victims, so extensive preparation and training are necessary^12^. Several studies have shown that when health students receive training on violence, their awareness and ability to spot a victim and help within the limits of their competence increases^8,13,14^. In Estonia, midwifery students’ attitudes towards VAW have not been studied before, which is why this pilot study is necessary to find out the primary results and, based on this, future actions. The purpose of the study is to investigate the attitude of midwife students towards VAW.

## METHODS

### Study design

This pilot study followed a mixed-methods approach, which is necessary for this research as it incorporates several methods to address appropriate and fundamental research questions involving collecting, analyzing, and interpreting qualitative and quantitative data^15^. Thus, qualitative and quantitative data are collected and analyzed simultaneously^16^. The pilot study is used to validate the questionnaire and obtain initial information. Polit and Beck^17^ emphasized that a pilot study evaluates the adequacy of the proposed method and procedures.

### Subjects

The pilot study population consisted of all 77 midwifery students at Tallinn Health Care College from 2022–2023. In Estonia, the duration of the midwifery curriculum is 4.5 years and a Bachelor’s degree is acquired. All respondents were female and aged 19–37 years. The online questionnaire was sent to all the midwifery students. Everyone had an equal opportunity to respond to the questionnaire, and 48 (62%) of the midwifery students completed the online survey.

### Ethics

To carry out the research, the Tallinn Health Care College (No. 1-16/238; 21.04.2020) and The National Institute for Health Development Research Ethics Committee of Estonia (No. 2725; 19.04. 2020) provided approval. All study data were collected using an electronic survey and stored on a private, password-protected computer accessible only by the principal investigators. The subjects were informed about the importance of this study and that they had the right to refuse participation in the study at the stage of answering the questionnaire.

### The online survey

The authors prepared the online survey based on the basic model of attitudes (knowledge, beliefs, behavior). The online questionnaire was created in the Connect environment, an online survey platform (https://www.connect.ee). Attitudes are based on a person’s knowledge, beliefs, and, as a result, behavior presented in relation to a phenomenon or a person^18^.

The online survey consisted of 23 questions structured into three topic blocks. The study contains 17 items to ask only the most representative questions, and questions the respondents did not clearly understand were left out. There are questions where the respondent can select multiple answer options, and only one answer option can be selected. The first topic block contains four open questions on types of VAW and the respondent’s exposure and/or experiences with them. The second topic block is based on a 5-point Likert scale^19^. On the Likert scale, statements are scored from 1 to 5 (5 = ‘do not agree at all’, 4 = ‘do not agree’, 3 = ‘cannot say’, 2 = ‘mostly agree’, and 1 = ‘completely agree’). The questionnaire contains eight questions about respondents’ sociocultural misconceptions related to VAW. The third topic block contains one open question about knowledge about the impact of violence on women’s health, one open question about VAW-related legislation in Estonia, and four open questions about future midwife’s awareness and possible skills in assisting the victim. Respondents could give additional thematic comments to all questions.

### Analysis

For the quantitative data of the questionnaire, relative frequencies (%) are presented. The mean, standard deviation, and minimum/maximum were calculated based on the Likert scale scores. Stata 14 was used for quantitative analysis. Qualitative data are used in this article to illustrate the results with comments and are not analyzed separately.

## RESULTS

The results of the first topic block show that respondents know different types of VAW. The respondents of this study have personal experiences and exposures to different types of violence (Table 1). Respondents admitted that they had experienced mental (emotional) VAW. The most common form of emotional VAW that respondents see or experience is commanding at 13%, commenting on appearance at 13% and nagging at 12%. Among the forms of mental (emotional) violence they experienced or saw, the respondents also mentioned inducing guilt 11.6%, threatening and mocking 11.3%. Remarks on mental abilities were also mentioned by 6.8%.

**Table 1:** Experiences and exposures of midwifery students, aged 19–37 years, to different types of violence, Tallinn Health Care College, 2022–2023 (N=48)

*Mental (emotional) violence*	*%*
Commanding	13.0
Appearance (body weight, clothing, etc.)	13.0
Nagging, scolding	12.0
Inducing guilt	11.6
Threatening and mocking	11.3
Remarks on mental abilities	6.8
**Physical, including sexual violence**	
Pushing	18.3
Pinching	14.5
Pulling (hair, clothing)	14.5
Touching or groping the intimate area	14.5
Forcing sexual intercourse (rape)	9.7
Hitting with the hand	8.1
**Economic violence**	
The partner (life partner) controls economic expenses	27.8
The partner (life partner) does not contribute materially to the joint life	24.1

*Examples of comments:*

*‘My dad was constantly bossing and yelling at my mom and me, and it was so destructive.’*

*‘I have seen many times how men mock or threaten their wives or partners.’*

*‘Constantly making comments about a woman’s appearance is so humiliating. Body weight, clothes, general appearance are criticized … well, whatever.’*

*‘The constant emotional abuse has destroyed my self-esteem.’*

The respondents’ experiences or exposure to physical violence (including sexual violence) are as follows. The most frequent type of physical VAW seen or personally experienced by the respondents has been pushing 18.3%, pinching 14.5%, pulling (hair or clothes) 14.5% (touching or groping the intimate area 14.5%, forcing sexual intercourse (rape) 9.7%, hitting with the hand 8.1%).

*Examples of comments:*

*‘He hit and pushed me with both hands at the same time, causing me to fall down.’*

*‘Several times, I saw a man grab a woman by her hair or her hand and drag her away.’*

*‘I myself have experienced all kinds of physical violence, I have also seen how women are squeezed, dragged, and hit by hand.’*

*‘At a party, while dancing, a man has touched me in the intimate area, and he found it to be normal’.*

The form of economic violence the respondents have encountered is when the partner (life partner) controls economic expenses 27.8% or the partner (life partner) does not contribute materially to the joint life 24.1%.

*Examples of comments:*

*‘My partner paid the apartment loan, and I paid for food, but when we break up, he has a home, I have neither a home nor money.’*

*‘My partner systematically monitors what I buy and how much I spend. Of course, he himself did not contribute.’*

The results of the second topic block show respondents’ sociocultural misconceptions related to VAW. The most telling examples are presented in the analysis, but not all questions and comments are on the same topic.

Respondents were presented with a statement to which they could respond using a Likert scale from 1 to 5, where number 5 represented ‘do not agree at all’, number 1 represented ‘completely agree’, and number 3 ‘cannot say’ (Table 2). Respondents also had the option to clarify their answers in words or add a comment.

**Table 2:** Scores of statements on sociocultural misconceptions related to VAW of midwifery students, aged 19–37 years, Tallinn Health Care College, 2022–2023 (N=48)

*Statements*	*Mean*	*SD*	*Min*	*Max*
He who beats, loves	4.9	0.14	4	5
Being hit once is not yet violence	4.8	0.49	2	5
If a woman nags, a man can hit her	4.9	0.40	3	5
A woman’s place is mainly at home (in the family)	4.6	0.73	2	5
Wearing a short skirt and/or a wide neckline is one of the causes of sexual violence	4.8	0.44	2	5
If during a sexual act a woman wants to interrupt (give up) the act and the man refuses, it is rape	1.5	0.85	1	4
Educated men are not violent	4.4	0.69	3	5
The woman is to blame herself if the man has hit her	4.8	0.44	3	5

VAW: violence against women.

In general, respondents were unanimous regarding the given statements. The most agreement was with the statements: ‘He who beats, loves’ and ‘if a woman nags, a man can hit her’, for which ‘do not agree at all’ was rated on average at 4.9 points.

There was slightly less consensus on the statements: ‘Being hit once is not yet violence’, ‘Wearing a short skirt and/or a wide neckline is one of the causes of sexual violence’, and ‘The woman is to blame herself if the man has hit her’. Respondents generally disagreed with these statements, rating them on average at 4.8 points.

One of the respondents shared his opinion on ‘Wearing a short skirt and/or a wide neckline is one of the causes of sexual violence’ by commenting:

*‘A woman and her clothes are never to blame for being used.’*

Another respondent had a similar point of view and comments:

*‘You might as well be sexually assaulting men on the beach because they’re only wearing swimming trunks. Just as well, men in the gym could be sexually abused because many of them are physically very good-looking and wear tight or short workout clothes. The same person understands that another person can be naked, but he still doesn’t abuse him.’*

However, one respondent disagreed with that point of view and believed that women often provoke perpetrators of violence with their clothes:

*‘I am disgusted because quite often women themselves provoke men and later complain that they are victims of sexual violence.’*

In general, it was found that respondents also disagreed with the statement ‘A woman’s place is mainly at home (in the family)’, rating it on average at 4.6 points. One respondent commented on this:

*‘It depends on how the family divides responsibilities and roles. If the woman wants to go to work and the man to be at home, then you can find an option to live just like this. “A woman’s place is at home” is quite an outdated expression. People’s pace of life has become faster, people are socially more active. However, if a partner with such a mindset keeps a woman at home, it is a violation of the woman’s rights and abuse.’*

Most respondents agree with the statement ‘If during a sexual act, a woman wants to interrupt (give up) the act and the man refuses, it is rape’, rating this statement on average at 1.5 points. Respondents commented:

*‘It’s so hard to prove, it’s word for word; It’s better not to let things go that far. Does the woman even have any choice in this situation?’*

Although respondents generally ‘do not agree’, at the same time, most disagree with the statement ‘Educated men are not violent’, rating it on average at 4.4 points.

Three respondents commented:

*‘Educated men are often the most violent because they usually bring stressful work and think they can take advantage of their position. I have observed that especially such older, educated men are mentally oppressive because they take advantage of their position. I have personally experienced it.’*

*‘Educated men often have higher self-esteem and therefore think everything is allowed to them.’*

*‘Violence occurs among people with every level of education; a higher level of education perhaps only makes people more cunning, how to hide their violence from the rest of the world and do it in front of others pretending to live an ideal family life.’*

The third topic block contains questions about knowledge of the impact of violence on women’s health, VAW-related legislation in Estonia, and the future midwife’s awareness and possible skills in assisting the victim. The third block used open questions. Knowledge of the impact of violence on women’s health was asked as an open question, asking to name various factors (Table 3).

**Table 3:** Awareness of the impact of violence on women’s health among midwifery students, aged 19–37 years, Tallinn Health Care College, 2022–2023 (N=48)

*Impact factors*	*%*
Infertility	17.6
Mental health issues	10.3
Miscarriage	9.6
Unwanted pregnancy	8.1
Sexually transmitted infections	6.6
Hormonal changes and menstrual cycle disorders	6.6
Physical and reproductive organs injuries	6.6
Bleeding	5.1
Premature birth	4.4
Abortions	2.9
Manipulation of contraception	2.2
Risky behavior (alcohol, drug use)	2.2
Pain	1.5

To the open question: ‘Is VAW punishable by law in the Republic of Estonia?’, 46.6% of respondents answered ‘Yes’, 12.8% answered ‘Do not know’, and 40.6% expressed that there is no law specifically regarding VAW in the Republic of Estonia.

*Examples of comments:*

*‘Certainly, acts related to some kind of violence are criminally punishable, but I suggest that the Penal Code does not recognize a separate concept of violence against women.’*

*‘Yes, but unfortunately, only physical and very severe violence can be punished in court.’*

*‘We have all kinds of anti-violence strategies in society, but women as victims continue to be unprotected in many ways.’*

An open question asked about the future midwife’s awareness and possible skills in assisting the victim. Theoretical knowledge about violence was considered necessary by 27.3% of the respondents. How to empathically communicate with the victim, understand, support, and help her was considered important by 28.2% of respondents, 27.3% of respondents considered the right intervention (marking, examination, victim assistance, network cooperation, legislation, etc.) necessary, and 48% of the respondents stated that they could not properly help the woman as a victim.

Examples of comments:

*‘My knowledge and skills are incomplete, I would like to be able to help the victim, but so far, I have not received such knowledge or practice.’*

*‘Correct handling of violence against women is a long process, both theoretical knowledge and practical experience are needed in order to properly provide help.’*

*‘I know one thing or another, but that’s not enough to help the victim.’*

In response to whether the midwifery curriculum deals with topics related to violence sufficiently, 63.8% of respondents found that the midwifery curriculum does not cover topics related to violence to the necessary extent.

*Examples of comments:*

*‘Violence against women is currently covered in only one subject and very few.’*

*‘There could be more hands-on training and simulations to develop skills.’*

*‘The topic of violence should be covered throughout the curriculum, not just as one topic in one subject.’*

*‘To be honest, topics about violence should be mandatory in the curriculum because with this we can reduce violence against women in our culture. In addition, the situations in the simulation must be dealt with, how to advise the woman, and offer the best help.’*

## DISCUSSION

VAW is a significant problem in both Estonia and worldwide. In Estonia, the attitudes of midwife students toward VAW have not been studied before. Several studies have contributed to the study of VAW in the last decade, but the attitude of midwife students toward VAW has been studied to a limited extent.

The results show that the respondents of this study have personal experiences and exposures to different types of violence. The most common form of emotional VAW that respondents see or experience is commanding and commenting on appearance (body weight, clothing, etc.). The respondents’ comments express that mental violence has a destructive effect and damages self-esteem. Similar studies in Turkey found that students studying in the health curriculum had experienced mental VAW that had affected them. Mental violence arose mostly from a male family member or loved one^9,20,21^. We believe that experiencing mental violence is a serious problem and can affect an individual’s ability to cope for a long time, and that the manifestations of mental violence are probably more extensive than the respondents expressed. There is reason to assume that different forms of mental violence cannot be distinguished or that some manifestations are so accustomed that they are not even noticed. The Pettai and Kase^3^ Estonian study showed that, according to healthcare workers, the biggest form of violence is mental violence against women, and this is in 82% of the cases. Mental violence seen or experienced personally, either in childhood or later in life, affects various aspects of an individual’s life^22^.

The most frequent type of physical VAW seen or personally experienced by the respondents has been pushing, but there are also other manifestations of physical (including sexual) violence. A study conducted by Pettai^23^ in Estonia in 2022 shows that 54% of women have experienced some form of physical violence, but there is a widespread opinion in society that VAW is overemphasized. The position of the study is that midwifery students are both members of society and future healthcare workers, so it is unfortunate that they have experienced physical (including sexual) violence. Several studies show that female students who have experienced violence, including physical violence, reported that it affected their academic results as well as their self-esteem^24-26^. Pereira et al.^26^ emphasize that any VAW affects a woman’s ability to cope with self-esteem and is a serious threat to mental and physical health.

Economics is not the first association that comes to mind when thinking or talking about VAW^26^. Respondents’ experiences with economic violence involved with current or previous life partners related to expenditure control or economic non-contribution. The FRA survey^27^ sees economic violence as part of psychological violence but separates it, matching the concept with the following behavior on the part of the respondent’s partner: ‘Preventing the respondent from making decisions on family finance or shopping independently, or forbidding her to work outside the home’. Bettio and Ticci^28^ note that economic VAW limits women’s freedom and independence. An economic VAW suppresses a woman’s self-esteem, decision-making ability, and access to education and the labor market^29^. Although the prevalence of economic violence among students is estimated to be low, even lower than that of working women, it has a psychological impact and negative consequences^28^. Some studies confirm that healthcare professionals who have experienced violence themselves are more supportive and empathetic towards victims^9,23^. Based on the Hegarty et al.^7^ study, more than 20 scientific studies show that a health worker’s personal experience of violence has a greater impact on the victim’s commitment, understanding, and willingness to help.

The results of the second topic block show respondents’ sociocultural misconceptions related to VAW and their knowledge about the impact of violence on women’s health. Over 90% of midwife students do not agree or do not agree at all with the negative myths and stereotypes prevalent in society regarding VAW. We believe that this is a positive result, as a negative attitude towards stereotypes in society is an effective potential for helping and supporting the victim of violence. At the same time, we emphasize that even a small percentage of the sociocultural misconceptions that appeared in the study need attention and changing attitudes. The literature has largely shown that myths of violence are strongly linked to the support of gender stereotypes and ambivalent sexist attitudes that contribute to the prevalence and perpetuation of VAW^13,30,31^. Social myths and negative stereotypes about abused women encourage the justification of violence^13^.

Respondents’ awareness of the impact of violence on women’s health is limited. The respondents did list various risks to a woman’s health caused by violence, but the percentage representation of specific risks is quite limited by the respondents. Undoubtedly, the theoretical knowledge and practical experience of the students in the first years is less than the knowledge of the students in the last years. The midwifery curriculum of the Tallinn Health Care College includes VAW as part of the study subject of Women’s Diseases (4 ECTS), but generally focuses on helping victims of sexual violence. Hence, students’ knowledge about VAW is limited. Several representative studies confirm the negative impact of VAW on women’s mental and physical health^32-34^; therefore, midwifery students’ knowledge of the impact of violence on women’s health is crucial. Lack of or limited knowledge of VAW among future healthcare workers is an obstacle to helping victims^35^.

Less than half of the respondents of this study are aware that VAW is punishable by law, which shows that midwifery students are not sufficiently familiar with the legislation in force in Estonia. We are convinced that knowledge of legislation must be represented in the curriculum, it is a prerequisite for providing help to victims of violence. Less than half of the respondents stated that they would not be able to provide proper help to the woman as a victim, as they lack knowledge, skills, and experience, which is an unfortunate factor. The Simsek and Ardahan^20^ study found that final year nursing and midwifery students can recognize the signs of VAW but lack the skills to help the victims. Several authors also consider lack of knowledge an important factor, recognized as the most important barrier to care, inquiry, and diagnosis of VAW by healthcare professionals^5,35^. The authors of the current study believe that topics related to violence need to be addressed in the midwifery curriculum from the first year of study until the end of the study period, giving students both knowledge and practical skills to provide the best possible help to the victim. Several authors agree that undergraduate courses are the ideal means to change nursing and midwifery attitudes toward VAW, as undergraduate courses can equip students with a comprehensive understanding of VAW. Undergraduate midwifery and nurse education must continually stress the relationship between exposure to violence and poor health; this will allow students to recognize exposure to violence and respond appropriately when they are treating patients in clinical settings in the future. There is strong evidence that violence training increases confidence and skills in aiding a victim of violence in a clinical setting^8,12,36^. We emphasize that to change attitudes, provide broad-based knowledge, and gain practical experience, it is necessary to introduce qualitative changes in the midwifery curriculum to ensure the best possible help for victims of violence. Several studies confirm that evidence-based theory learning, simulations, workshops, etc., could be suitable methods. Therefore, the subject of violence should be included in the curricula by integrating it into existing courses or as a separate course in each academic year^12,31^. Health educators should be strongly encouraged to provide midwifery students with additional simulations, collaborative activities, and theory training on violence, to gain practical experience in dealing with VAW.

### Limitations

The present pilot study has several limitations. The main limitation was the small number of respondents, which does not allow for statistical generalizations or connections, but allows for a primary analysis of midwife students’ attitudes toward VAW and to notice the bottlenecks that have arisen. An important drawback is that the study did not distinguish students by year of study, so students’ actual knowledge in terms of WAV is not known exactly. Obviously, the knowledge of final-year students may differ from that of first-year students. The limitations arising from the questionnaire should be noted. Some of the questions were either misunderstood or were not formulated in the best way, which resulted from the peculiarity of the Estonian language. Although these questions were not considered in this study and were left aside, their better wording would have ensured the quality of the study. The wording of the questions needs to be corrected, reformulated and made clearer to avoid multiple interpretations. Nevertheless, it is worth noting that the in-depth comments of the respondents made it possible to provide the first important insight into the researched topic, enabling improvement of the bottlenecks of the midwifery curriculum in terms of VAW, both in theory and practice.

## CONCLUSIONS

The present pilot study showed that midwife students’ attitudes towards VAW need improvement. Topics related to violence should be included in the midwifery curriculum, along with the development of practical skills. In the midwifery curriculum, violence-related issues need to be addressed from the first year of study to the end of the course, giving students both the knowledge and practical skills to provide the best possible care to victims. The present pilot study raises the need to investigate midwife students’ attitudes towards VAW with a larger sample, better-designed method, and validated questionnaire.

## Data Availability

The data supporting this research are available from the authors on reasonable request.
